# Differential Analysis of Sesquiterpenoids of *Atractylodes macrocephalus* from Different Origins Based on Transcriptomics

**DOI:** 10.3390/molecules31071075

**Published:** 2026-03-25

**Authors:** Ao Sun, Xin Yu, Shan Lu, Tong Wu, Ke-Yi Meng, Jing-Wei Hao, Nan Zhao, Jun-Hong Chai, Ting-Ting He

**Affiliations:** 1 College of Life Science and Technology, Mudanjiang Normal University, Mudanjiang 157011, China; sunao9215@126.com (A.S.);; 2 Department of Pharmaceutical Engineering, School of Biomedical and Pharmaceutical Sciences, Guangdong University of Technology, Guangzhou 510006, China

**Keywords:** *Atractylodes macrocephala*, transcriptome, differentially expressed genes, sesquiterpenes

## Abstract

*Atractylodes macrocephala* (A.M.) is a traditional Chinese medicinal and edible herb renowned for its spleen-tonifying, dampness-resolving, diuretic, and antiperspirant properties. Its primary bioactive constituents are terpenoids, which have demonstrated anti-inflammatory, antitumor, and immunomodulatory activities. However, transcriptomic studies focusing on terpenoid biosynthesis in A.M. from different geographical origins remain limited. To investigate the molecular mechanisms underlying differential sesquiterpenoid production, we performed transcriptome sequencing on samples collected from four distinct regions in China. Sesquiterpenoid biosynthesis predominantly proceeds through the mevalonate (MVA) and methylerythritol phosphate (MEP) pathways. Comparative analysis revealed four key enzyme-encoding genes—*HMGCR*, *ISPF*, *GCPE*, and *FDPS*—whose differential expression patterns were further validated by quantitative real-time PCR (qRT-PCR). Samples from Shaanxi exhibited the highest upregulation of biosynthetic genes and the greatest enrichment of terpene-related metabolites, suggesting enhanced pharmacological potential. In contrast, samples from Fujian, Anhui, and Hebei displayed relatively lower activity, with only *FDPS* upregulated in the Hebei sample. High-performance liquid chromatography (HPLC) quantification confirmed regional differences in the levels of major terpenoids—including atractylodin, atractylenolide I, and atractylenolide III—which correlated well with the observed gene expression profiles. This study compared conspecific A.M. from different geographical regions and further revealed that the variation in terpenoid metabolites is closely related to environmental factors. These findings provide a theoretical basis for the further discovery of functional genes and offer important implications for the quality control of A.M.

## 1. Introduction

*Atractylodes macrocephala* Koidz (A.M., Baizhu in Chinese) is a perennial herb belonging to the genus *Atractylodes* of the family *Asteraceae*. It prefers mild and cool climates, and wild plants are distributed in mountainous regions, hills, forests, and scrublands at altitudes of 1000–1800 m [1]. Its rhizome is the central medicinal part, with multiple medicinal uses. It was utilized as early as the Tang Dynasty in China and was spread to Japan in the 18th century; since then, it has been widely cultivated not only in Japan but also in neighboring countries such as South Korea and the Far East of Russia, where A.M. is also commonly used in traditional medicine systems. For instance, in Japan (where it is called “Byakujutsu”), the rhizome of A.M. is a common ingredient in Kampo medicines, such as Banhabaekchulchunma-tang [2], and is also used in folk prescriptions to alleviate dizziness and edema [3]. It is widely recognized as a common digestive and tonic herb in traditional Korean medicine, and is recorded in ancient medical works such as the “*Hyangyak Jipseongbang*” for its effects in tonifying the spleen and regulating gastrointestinal function [4]. In the Far East of Russia, it is often used in folk medicine to relieve fatigue.

In China, it is primarily distributed in the regions of North, Central, and South. In addition, it is also widely cultivated in Japan, Korea, and the Far East of Russia. It also has many alternative names, such as Yu Zhu, Dong Zhu, Zhe Zhu, and Shan Lian. It is a well-known warming and tonifying herb in traditional Chinese medicine [5]. The earliest record of it can be found in “Shen Nong’s Materia Medica”, where it is classified as a superior quality herb and is considered non-toxic [6]. In traditional Chinese medicine, it is considered to have a bitter and sweet taste and to be warm in nature, which theoretically corresponds to its therapeutic actions of invigorating the spleen, replenishing qi, and drying dampness—making it traditionally used for conditions such as poor appetite, fatigue, and diarrhea associated with spleen-qi deficiency [7]. A.M. is well-recognized for its ability to both strengthen the spleen and harmonize the stomach. Modern pharmacological studies have demonstrated that this stomach-harmonizing effect is associated with gastrointestinal regulatory activities, including promoting gastric emptying, protecting the gastric mucosa, and exerting anti-inflammatory actions in the digestive tract [8,9].

Research on the accumulation of secondary metabolites in medicinal plants has a long history. Previous studies have demonstrated that the biosynthesis and accumulation of bioactive compounds are affected by a combination of genetic, developmental, and environmental factors, leading to significant differences in secondary metabolite profiles among different plant organs and geographical regions [10]. Similarly, regional differences in climate, soil composition, and related biological factors have long been regarded as key determinants of chemotypic diversity in medicinal plants [11]. In recent decades, a wide variety of chemical constituents have been isolated from A.M., namely, volatile oils, polysaccharides, lactones, flavonoids, and others [12,13,14]. The terpenoids of A.M. include volatile oils, atractylenolides I~IV, and various lactones, all of which possess potent antioxidant activity [15,16,17]. Importantly, sesquiterpenoids are currently regarded as the most important therapeutic compounds in the rhizome of A.M. and the compounds in question are mainly found in the volatile oil of A.M. [18]. These compounds exhibit abundant pharmacological properties, including anti-inflammatory, anticancer, and organ-protective effects, and are potent inhibitors and modulators of inflammation [19,20]. More importantly, differences in gene expression regulation arising from different geographical origins result in variation in both the content and the types of sesquiterpenoids present in A.M. medicinal materials [21,22,23]. However, the molecular mechanism underlying sesquiterpene accumulation has not yet been fully clarified, particularly with respect to the biosynthetic pathways and regulatory mechanisms of its bioactive compounds. The insufficient integration of transcriptomic data with corresponding product quantification data (e.g., HPLC) hinders the systematic elucidation of regulatory networks governing the accumulation of specific chemical constituents.

Transcriptomics uses whole-genome sequencing to study RNA expression, which is an important method to understand cell phenotypes and functions [24]. Transcriptomes have been applied to investigate biosynthetic pathways of bioactive compounds [25]. Current research on A.M. primarily focuses on its overall efficacy, component analysis, and network pharmacology mechanisms [26,27]. Existing transcriptomics research predominantly focuses on the regulatory effects of its extracts on the gut microbiota [28,29,30]. However, its secondary metabolic pathways and metabolic processes remain unclear, limiting research on the synthesis of active components. Additionally, comparative studies on the pharmacological effects of A.M. across different regions are relatively scarce.

This study employed transcriptomics to analyze differentially expressed genes (DEGs) in A.M. at the same growth stage across different origins and screened for DEGs associated with sesquiterpene compound biosynthesis and accumulation. To validate transcriptomic results and metabolite accumulation, key sesquiterpene compounds were quantified using high-performance liquid chromatography (HPLC). The study jointly validated the mechanisms by which geographical variation influences the formation of bioactive compounds in A.M. at both transcriptomic and metabolic levels, providing a scientific basis for molecular breeding, functional gene identification, and active component research on this species.

## 2. Results and Analysis

### 2.1. Transcriptome Data Assembly

The cDNA libraries of A.M. rhizomes from four different origins were sequenced and constructed. The total number of raw reads was 48,741,824~76,311,598 bp, and the total number of clean reads was 47,615,740~74,287,334 bp. The average of Q20, Q30, and GC content was 98.75%, 95.27%, and 46.09%, respectively (Table A1).

We obtained 915,152 transcripts and 374,475 unigenes from transcript assembly (Table 1). Both datasets were predominantly composed of sequences longer than 500 bp, with 390,703 transcripts and 129,529 unigenes in this size range; furthermore, 174,160 transcripts and 49,105 unigenes exceeded 1000 bp. The average lengths of the transcripts and unigenes were 688.75 bp and 574.27 bp, respectively, with corresponding N50 values of 1003 bp and 760 bp, and N90 values of 760 bp and 256 bp. These metrics indicate a high-quality transcriptome assembly, characterized by a substantial proportion of medium- to long-length sequences, which are sufficient to support downstream analyses such as gene annotation, differential expression profiling, and the identification of key genes involved in sesquiterpenoid biosynthesis.

### 2.2. Unigene Annotation and Classification

**Figure 1:**
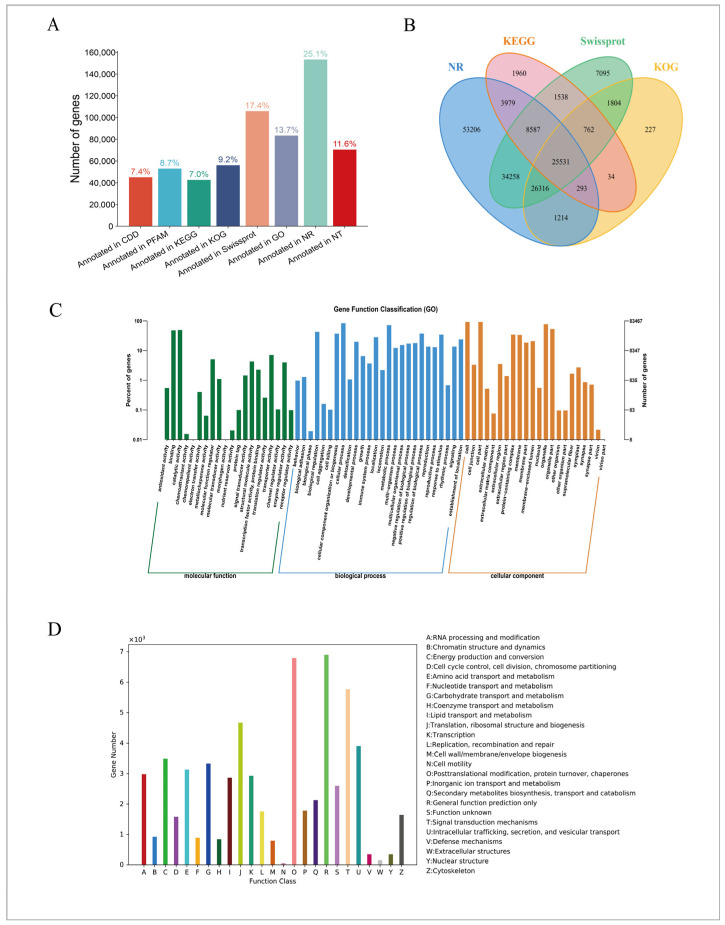
(**A**) Unigene annotation ratio by database. (**B**) Venn diagram. (**C**) Histogram of GO annotation distribution. The x-axis represents the secondary GO categories, and the y-axis shows both the number of genes within each category (right) and their percentage relative to the total number of annotated genes (left). Different colors represent different orthologs. (**D**) Histogram of the KOG classification. In the KOG classification, the assembled unigenes are categorized into 24 classes. The y-axis indicates the number of unigenes in the class, and the x-axis indicates the KOG classification.

The spliced unigenes were compared with eight commonly used databases, and 182,821 (48.82%) unigenes were annotated in at least one database. Among them, only 3485 (0.93%) unigenes were annotated in all databases. The NR database contained 153,384 annotated unigenes, which have a high proportion reaching 40.96%, while the KEGG database had 42,684 annotated unigenes, accounting for only 11.4% (Figure 1A). In addition, the number of genes annotated in the CDD, PFAM, KOG, Swissprot, GO, and NT databases was 45,077 (12.4%), 53,058 (14.17%), 56,181 (15.00%), 105,891 (28.28%), 83,467 (22.29%), and 70,545 (18.84%), respectively. Therefore, unigenes annotated in the four databases, NR, KEGG, Swiss-Prot, and KOG, were selected to make a Venn diagram (Figure 1B). The results showed that there were 25,531 unigenes annotated in the four databases, and 53,206 unigenes were annotated only in the NR database. In addition, there were only 1960 unigenes recorded in the KEGG database, 7095 unigenes annotated in the Swiss-Prot database, and 227 unigenes annotated in the KOG database.

A total of 1,048,575 unigenes were classified into three main functional categories: 60.54% were assigned to BP (biological process), 24.95% belonged to CC (cellular component, j cell components), and 14.51% were attributable to MF (molecular function) (Figure 1C). A total of 62,586 genes were annotated in KOG, which was divided into 25 classes (Figure 1D). Among them, general function prediction only (6899, 11.02%) represented the highest proportion, followed by posttranslational modification, protein turnover, and chaperones (6797, 10.86%), and signal transduction mechanisms (5774, 9.23%).

### 2.3. Different Origins Correlation Analysis Between Samples

**Figure 2:**
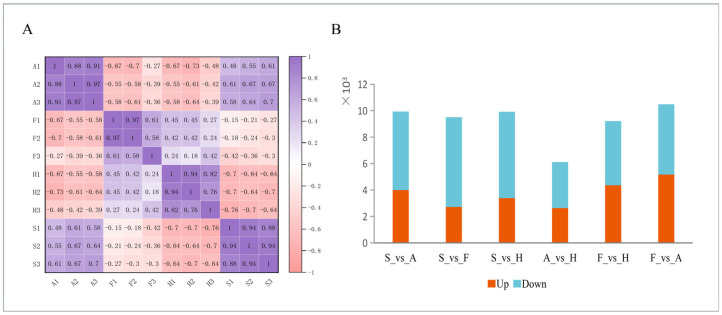
(**A**) Heatmap of pairwise correlation coefficients between samples. The color scale ranges from −1 (pink) to 1 (purple), with darker colors indicating higher correlation. (**B**) Number of significantly differentially expressed genes (DEGs) in six pairwise origin comparisons. Red bars stand for upregulated genes, and blue bars stand for downregulated genes. DEGs were defined as |log_2_(fold change)| ≥ 1 and adjusted *p*-value < 0.05.

To assess reproducibility across the collected samples, three biological replicates were tested on A.M. rhizomes from four different origins: Anhui (A1, A2, A3), Fujian (F1, F2, F3), Hebei (H1, H2, H3), and Shaanxi (S1, S2, S3). The results revealed that, in this study, the correlation coefficient between samples from the same tissue was at or above 0.8, suggesting that reproducibility between sites within the same tissue was relatively good, with no outliers (Figure 2A).

### 2.4. Differential Expression Analysis

DESeq was used to analyze DEGs, which were derived from 12 samples, and the screening conditions were *p*-value < 0.05 and |log_2_(fold change)| ≥ 1. In this situation, the expression patterns of A.M. samples were compared. According to the principle of differential gene expression screening, expression patterns of A.M. samples from different provenances were compared. Figure 2 illustrates the differential expression analysis of the six groups, showing that downregulated DEGs in all groups exceeded upregulated ones. The F vs. A group exhibited the highest total number of DEGs, while the A vs. H group had the fewest (Figure 2B). This indicates that the degree of transcriptional differences varies among different regions.

### 2.5. Gene Ontology Analysis of Differentially Expressed Genes

**Figure 3:**
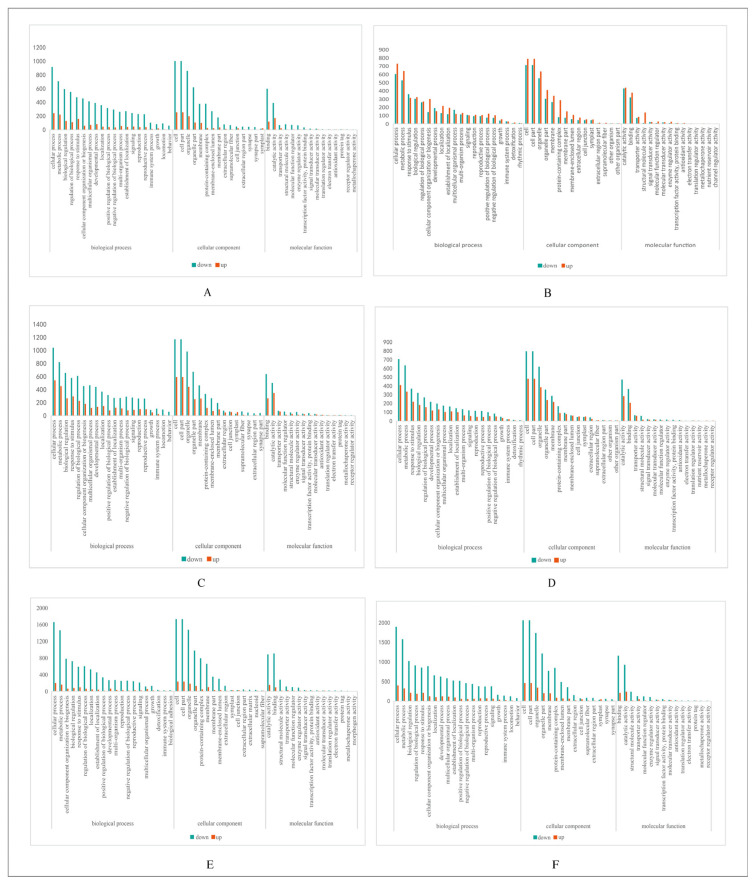
Classification of DEGs from the two origins by GO annotation. (**A**) A vs. H. (**B**) F vs. A. (**C**) F vs. H. (**D**) S vs. A. (**E**) S vs. F. (**F**) S vs. H.

To obtain the functional annotation of DEGs, the DEGs between the four origins of A.M. were mapped to the GO protein database and were divided into three categories: cellular component, molecular function, and biological process. The top 20, 15, and 15 GO terms were selected for presentation (Figure 3).

Analysis revealed that the functional distribution of DEGs displayed obvious differences among samples from different origins. Among biological process categories, “cellular processes” were the most significantly enriched term across all comparison groups, suggesting that regional differences primarily manifest at the level of fundamental cellular activities. Conversely, the entries with the fewest annotations varied across different origin comparisons, involving specific functions such as “behavior” and “rhythmic processes”, indicating that A.M. from different origins exhibits differentiated characteristics in specific physiological pathways. Regarding cellular components, “cell” and “cell part” were the most highly annotated entries, indicating that differentially expressed genes are mainly associated with basic cellular structures. Most cellular activities occur in diverse subcellular compartments [31]. This suggests that to adapt to the environments of different production areas (such as temperature, soil, and pH), A.M. not only alters genes in a single organelle, but instead exhibits changes in gene expression across the entire cell, involving a wide and comprehensive range of genes. The least annotated entries exhibited greater heterogeneity, including “symplast” and “synapse part.” This may indicate that A.M. from different origins exhibits differentiation in specific, intricate cellular structures or specialized functions. Within molecular function categories, “binding” or “catalytic activity” were the most enriched functions, indicating that metabolic and regulatory processes are influenced by geographical factors. Conversely, the least annotated functional categories exhibited certain inter-group specificities, possibly related to metabolite or regulatory mechanisms unique to certain origins.

### 2.6. Kyoto Encyclopedia of Genes and Genomes Metabolic Pathway Analysis of Differentially Expressed Genes

**Figure 4:**
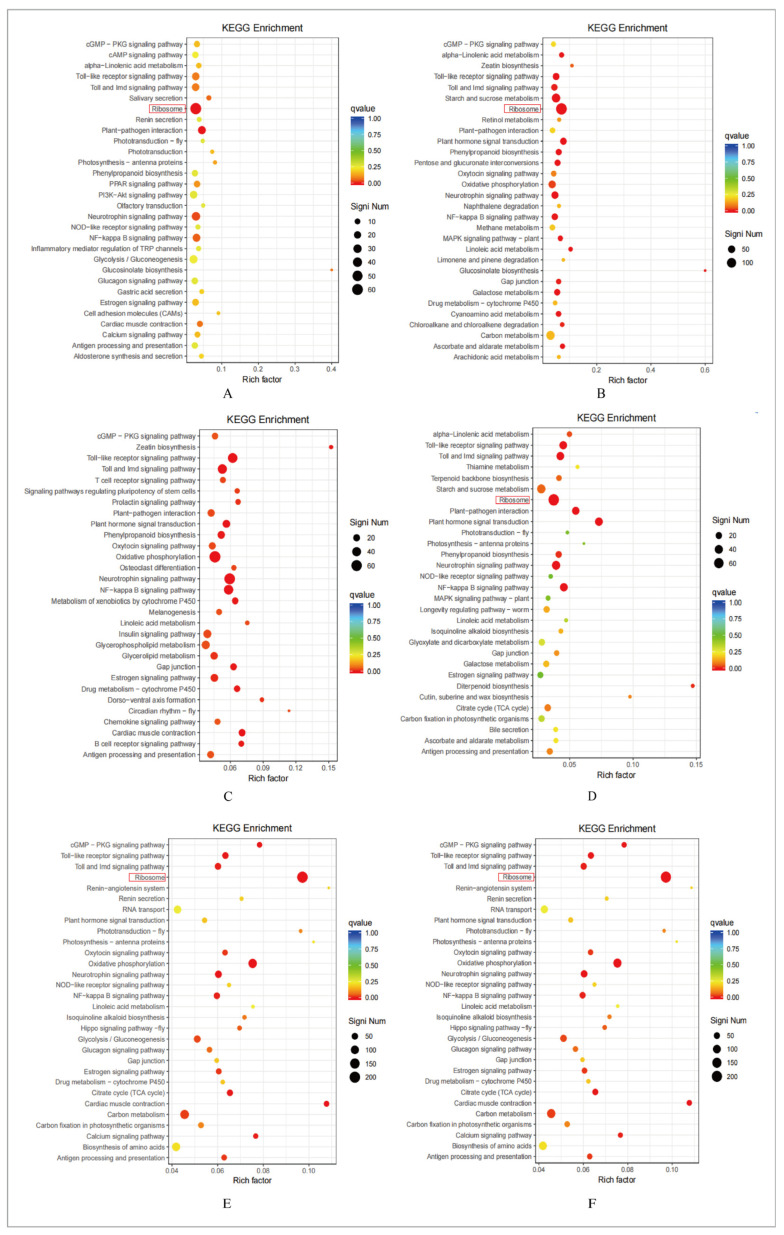
KEGG enrichment analysis results of DEGs in A.M. samples from different origins. The color gradient of the bubbles reflects the q-value, while the bubble size represents the number of enriched genes. (**A**) A vs. H. (**B**) F vs. A. (**C**) F vs. H. (**D**) S vs. A. (**E**) S vs. F. (**F**) S vs. H.

To further elucidate the biological functions of these differentially expressed genes, KEGG metabolic pathway enrichment analysis was conducted on DEGs from A.M. samples of the four origins. In the KEGG enrichment analysis, all DEGs were classified by q-value from high to low, and the top 30 items were selected to present the bubble map (Figure 4). The six groups of annotated DEGs related to the ribosome pathway were the most numerous, and DEGs related to carbon metabolism were also among the top five. Table 2 presents the top three types of metabolic pathways between A vs. H, A vs. F, A vs. S, H vs. F, H vs. S and F vs. S. The results revealed that the DEGs related to the ribosomal pathway were the most numerous in the six groups, and the DEGs related to carbon metabolism were also among to the top five.

The ribosome is the core organelle for protein biosynthesis. Significant enrichment of the ribosome pathway indicates strong translational activity, which promotes the synthesis of key enzymes involved in sesquiterpenoid biosynthesis. This provides a sufficient protein basis for the efficient synthesis and accumulation of sesquiterpenoids. Meanwhile, carbon metabolism, as a vital pathway for energy and precursor supply, acts coordinately with the ribosome pathway, which is closely associated with terpenoid accumulation in A.M.

### 2.7. Integrated Analysis of Terpenoid Biosynthesis Transcriptome

The main effective medicinal ingredients of A.M. are terpenoids, including atractylodin and atractylenolide. Its biosynthesis proceeds first via the general terpenoid skeleton biosynthesis pathway, followed by the sesquiterpene synthesis pathway [32]. The results are shown in Table 3. Twenty-six DEGs were found in the terpenoid skeleton generic synthesis pathway. According to the DEGs, the functional annotation information in the database was compared with six groups, and this study mined 12 key enzyme genes that are differentially expressed in terpenoid biosynthesis pathways.

**Figure 5:**
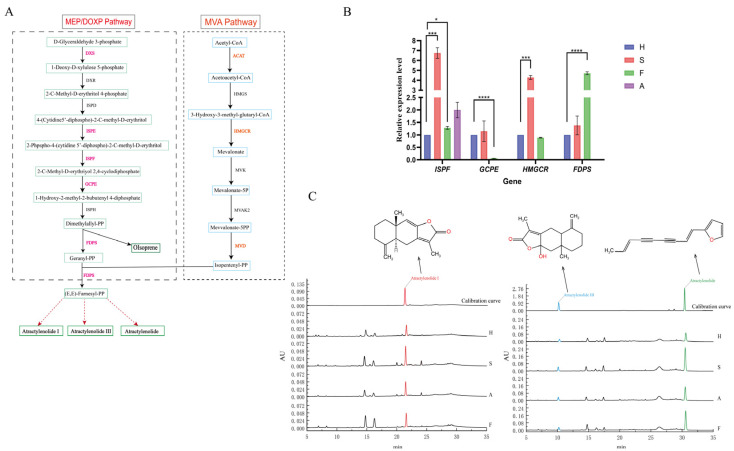
(**A**) Path diagram of the sesquiterpene biosynthesis pathway. Red and pink letters represent the key enzymes involved in sesquiterpene and triterpene biosynthetic pathways. Solid lines indicate direct catalytic reactions, and dotted lines indicate indirect catalytic reactions. (**B**) qRT-PCR analysis of four significant DEGs in sesquiterpene and triterpene biosynthetic pathways. The relative expression level of A.M. in Hebei was taken as the control. These values represent three biological repeats. * indicates significant (*p* ≤ 0.05); *** indicates highly significant (*p* ≤ 0.001); **** indicates extremely significant (*p* ≤ 0.0001). (**C**) The chemical structures of key metabolites and the chromatograms of A.M. root extract before and after stress obtained by HPLC analysis.

The sesquiterpenoids in A.M. are one of its main bioactive components, which have various pharmacological effects such as anti-inflammatory and antitumor activities. The biosynthesis pathway of sesquiterpenoids is mainly carried out through the mevalonate (MVA) and methylerythritol phosphate (MEP) pathways. In the MVA pathway, isoprenyl pyrophosphate (IPP) and dimethylallyl pyrophosphate (DMAPP) are condensed to form geraniol pyrophosphate (GPP), which is further condensed to form farnesyl pyrophosphate (FPP) and finally sesquiterpenoids (Figure 5A). Through transcriptome sequencing and bioinformatics analysis, eight key enzyme genes with differential expression in terpenoid biosynthesis pathways were obtained: *ACAT*, *HMGCR*, *MVD*, *DXS*, *ISPE*, *ISPF*, *GCPE*, and *FDPS*.

In this study, the genes with high upregulation were selected from the above key enzyme genes. *ISPF* or *GCPE* was screened from the MEP pathway, *HMGCR* was screened from the MVA pathway, and the key enzyme *FDPS* was screened for subsequent qRT-PCR detection.

### 2.8. Transcriptome Validation

To validate the results of transcriptome sequencing, four DEGs were selected to perform qRT−PCR analysis. The results are shown in Figure 5B; the level of the *ISPF* gene screened in the MEP pathway of A.M. in Shaanxi Province was significantly higher than that in other origins. The expression level of the *HMGCR* gene identified in the MVA pathway was significantly higher in Shaanxi Province. Moreover, the expression level of the *FDPS* gene, encoding a key enzyme, in A.M. samples originating from Fujian Province was significantly higher than that of other provenances. These results indicated that the expression levels of four key enzyme genes in the terpenoid biosynthesis pathway of A.M. in Shaanxi Province were relatively high, which was consistent with the results of transcriptome sequencing.

### 2.9. Correlation Between Terpenoid Compound Content

To further validate the consistency between gene expression and metabolic accumulation, HPLC was employed to determine the content of major sesquiterpene compounds (atractylenolide, atractylenolide I, and atractylenolide III) in A.M. from the four origins. The results showed that all three metabolites were detected in A.M. from all origins, though their contents varied (Figure 5C).

Specifically, the content of atractylenolide I was 4.06 ± 0.16 mg/g in Shaanxi, 2.54 ± 0.10 mg/g in Fujian, 2.42 ± 0.10 mg/g in Anhui, and 1.92 ± 0.08 mg/g in Hebei. The content of atractylenolide III was 4.71 ± 0.19 mg/g in Shaanxi, 3.80 ± 0.15 mg/g in Fujian, 3.38 ± 0.14 mg/g in Anhui, and 3.34 ± 0.13 mg/g in Hebei. The content of atractylodin was 18.29 ± 0.73 mg/g in Shaanxi, 14.93 ± 0.60 mg/g in Fujian, 18.29 ± 0.73 mg/g in Anhui, and 7.20 ± 0.29 mg/g in Hebei.

A.M. from Shaanxi exhibited the highest content of the main secondary metabolic components and the most significant metabolic accumulation, followed by samples from Fujian. In comparison, the contents in A.M. from Anhui and Hebei were relatively lower. The levels of terpenoid compounds in Shaanxi A.M. were higher than those in the other three origins. The accumulation pattern of sesquiterpenoids detected by HPLC was consistent with the high expression levels of key genes revealed by qRT-PCR, demonstrating that A.M. from Shaanxi possesses enhanced sesquiterpene biosynthetic capacity at both the metabolic and transcriptional levels.

## 3. Discussion and Outlook

In this study, transcriptomic analysis revealed the key regulatory genes in terpenoid biosynthesis in different producing areas of A.M. in Shaanxi, Fujian, Anhui, and Hebei. The results showed that the key genes of the sesquiterpenoid biosynthesis pathway (e.g., *HMGCR*, *DXS*, and *FDPS*) showed significant regional differences [33]. Among them, A.M. in Shaanxi Province showed synergistic upregulation of multiple genes in both MVA and MEP pathways, while A.M. in Hebei Province only had a higher expression level of *FDPS* genes. This result suggests that there were significant differences in the expression of MVA and MEP pathway genes in A.M. rhizomes from Shaanxi, Hebei, and other producing areas, and they were closely related to the content of sesquiterpenoids [34,35]. These findings are consistent with the traditional recognition of Shaanxi as a high-quality production area for A.M. and provide a molecular-level explanation for its superior medicinal efficacy [36,37].

Transcriptomic and secondary metabolic component analyses revealed that key biosynthetic genes (*HMGCR*, *DXS*, and *ISPF*) involved in the MVA and MEP pathways were differentially expressed in A.M. and significantly upregulated in Shaanxi. This synergistic upregulation indicates the coordinated activation of the two terpenoid precursor pathways, which elevates the cellular pool of common isoprenoid precursors (IPP and FPP) and promotes the accumulation of pharmacologically active sesquiterpenoids, including atractylenolide I, atractylenolide III, and atractylone [38]. Notably, high expression of the *FDPS* gene further drives the conversion of FPP to the sesquiterpene skeleton. These results indicate that the Shaanxi ecotype has an enhanced capacity for terpenoid biosynthesis [39]. The high expression of these rate-limiting enzymes in Shaanxi A.M. may be driven by the region’s unique ecological factors (e.g., diurnal temperature variation and soil trace element composition) through regulatory mechanisms at the transcriptional level [40]. Conversely, the relatively low expression of these key genes in non-authentic producing areas corresponded to reduced terpenoid accumulation [41]. Although the expression of *FDPS* in the Hebei production area is high, the sesquiterpene biosynthetic capacity is limited due to the insufficient expression of upstream genes such as *HMGCR* and *ISPF*. This regulatory imbalance of “precursor supply–skeleton formation” may be the key molecular mechanism of the low pharmacodynamic activity of A.M. in non-authentic producing areas [42,43].

Geographic differences in climate and soil properties may regulate sesquiterpenoid biosynthesis through epigenetic mechanisms, such as soil pH, organic matter, and mineral elements affecting plant metabolism and the activities of enzymes involved in secondary metabolism [44]. For instance, Xiong et al. [45] manipulated Dezhou (pH 8.43) and Jiangsu (pH 6.17) agricultural soils in China and found that soil pH affects bacterial communities far more strongly than fungal communities, with bacterial α-diversity following a quadratic pattern that peaked near each soil’s native pH; similarly, the soil pH varies among Shaanxi, Anhui, Fujian, and Hebei in our study, which may exert similar regulatory effects on microbial communities and subsequent secondary metabolism. Wang et al. [46] demonstrated that Fe^2+^ can significantly activates the MVA and MEP pathways involved in terpenoid metabolism in *Conyza blinii* and upregulates the expression of key enzyme genes, including *CbHMGR*, *CbFPPS*, and *CbDXS*. Essential elements from soil may act as cofactors or signaling molecules to regulate the transcription of terpenoid biosynthetic genes [47]. Studies have indicated that the soil microbiome is also involved in such regulation: rhizosphere bacteria in the genuine (“Dao-di”) production areas can directly or indirectly regulate the synthesis of secondary metabolites, which subsequently influences the “authenticity” of medicinal plants [48,49]; unique environmental conditions in the four study areas may further shape distinct rhizosphere microbial communities, thereby contributing to the regional quality differences of A.M. Thus, soil environmental factors are important external factors shaping the metabolic characteristics and quality of medicinal plants. Together, these factors form the “stress–defense–metabolism” axis, which promotes the high expression dominance of the genotype of A.M. in Shaanxi.

Long-term artificial breeding in authentic production areas may have screened for alleles optimizing sesquiterpene synthesis. Shaanxi A.M. may carry favorable SNPs in *HMGCR* and *ISPF* promoter regions. A similar artificial selection mechanism has been found in Panax ginseng, where the authentic genotype retains high expression of the *CYP716* allele [50,51]. In contrast, the short cultivation history of A.M. in Hebei may lack sufficient selection pressure to fix these alleles, resulting in its dependence on conservative *FDPS* expression.

Atractylenolide and other sesquiterpenes exhibit dual functions as photoprotectants and medicinal components. Mountain ecosystems in Shaanxi Province with high pathogen stress may drive defense-related genes (*HMGCR* and *FDPS*) to indirectly enhance medicinal efficacy [52,53,54]. This aligns with the “optimal defense” theory: the allocation of defense compound resources is positively correlated with the level of environmental threats [55]. Thus, the superiority of authentic medicinal materials may result from ecological adaptation and artificial selection.

These findings provide important guidance for the molecular breeding of A.M. First, breeding strategies should focus on the coordinated expression of multiple genes rather than overexpressing a single gene, especially the balance between precursor supply (e.g., *HMGCR* and *DXS*) and skeleton formation (e.g., *FDPS*). Second, molecular markers can be developed based on the superior genotypes from authentic regions, such as the highly expressed allelic variations in the Shaanxi population, to assist in the selection of elite germplasm with high terpenoid content. Furthermore, combined with the regulatory mechanisms of environmental response elements, future studies can use gene editing to optimize the promoter regions of key genes and simulate the expression patterns typical of authentic origins, thereby achieving targeted improvement of high-quality germplasm in non-authentic areas.

The transcriptome and metabolite analyses data generated in this study lay the foundation for the identification of key terpenoid regulatory genes, the functional verification of terpenoid biosynthetic pathways, and molecular marker-assisted breeding. This comprehensive multi-omics dataset also provides a valuable resource for future translational and applied research on medicinal plant breeding, geo-authentication, and quality improvement. As authentic medicinal materials, A.M. from Shaanxi displays clear advantages in the expression of key terpenoid biosynthetic genes. These findings provide a molecular basis for evaluating and controlling the quality of A.M., and also support the traditional authenticity concept of geo-authentic medicinal materials. Collectively, this study provides a novel perspective for understanding terpenoid accumulation and biosynthesis in A.M. and establishes a foundation for the selection and breeding of high-quality germplasm. We acknowledge certain limitations, including the sampling range and the need for further mechanistic validation. Nevertheless, with the development of multi-omics integration and intelligent breeding, it will become possible to reduce the traditional dependence of authentic medicinal materials on specific habitats. In addition, this study strengthens the hypothesis-driven framework and practical relevance by elucidating the molecular mechanisms underlying terpenoid biosynthesis and authentic medicinal herbs, thereby enhancing its application value in medicinal plant research.

## 4. Materials and Methods

### 4.1. Sample Collection

The plant material used in this study was collected from local planting areas in four cities of China: Ankang City, Shaanxi Province (32°68′ N, 109°02′ E); Bozhou City, Anhui Province (33°84′ N, 115°77′ E); Baoding City, Hebei Province (38°87′ N, 115°47′ E); and Quanzhou City, Fujian Province (24°91′ N, 118°58′ E), China. For each region, 6~8 individual plants were harvested and rhizome samples were obtained from three independent vendors, corresponding to the three biological replicates (S1–S3, A1–A3, F1–F3, and H1–H3). The plant samples were cleaned with water, drained on filter paper, and preserved in liquid nitrogen for further study. All plant materials were identified as A.M. by Dr. Shuang Yu, Mudanjiang Normal University.

### 4.2. RNA Extraction and Library Construction

Total RNA was extracted from four samples (S, A, F, and H) using the Total RNA Extractor (Trizol, Nanjing, China) kit following the manufacturer’s instructions: fresh samples were ground in liquid nitrogen, mixed with Trizol, incubated, and processed with chloroform and isopropanol sequentially, followed by ethanol washing, drying, and dissolving in RNase-free ddH_2_O. RNA quality, purity, integrity, and concentration were verified by agarose gel electrophoresis, a Qubit 2.0 Fluorometer (Invitrogen, Carlsbad, CA, USA), and an Agilent 2100 Bioanalyzer (Agilent Technologies, Palo Alto, CA, USA). Qualified samples were used to construct four cDNA libraries, which were then sequenced on the Illumina HiSeq2000™ platform (San Diego, CA, USA).

### 4.3. Evaluation of Sequencing Results and Transcript Splicing

Following sequencing, raw reads generated from the cDNA libraries were filtered to remove low-quality reads and adapters. The obtained clean reads were then used for statistical analysis of unigenes.

### 4.4. Functional Gene Annotation

The results obtained from unigene sequences using BLAST+ 2.14.0 software (National Center for Biotechnology Information, Bethesda, MD, USA) were compared with eight major databases, namely, NCBI protein non-redundant (NR), Kyoto Encyclopedia of Genes and Genomes (KEGG), Conserved Domains Database (CDD), Protein Families Database (PFAM), euKaryotic Orthologous Groups (KOG), Swiss-Prot Protein Sequence Database (Swissprot), Gene Ontology (GO), and Nucleotide Sequence Database (NT), to obtain unigene annotation information.

### 4.5. Analysis of Differentially Expressed Genes

The DEGs of A.M. in four different origins were identified by DESeq2 (Version 1.24.0) software. DESeq 2 provides statistical routines for determining differential expression in numeric gene expression data using a model based on negative binomial distributions. The DEGs were selected with the criteria of q-value < 0.05 as well as fold change |FoldChange| > 1. Venn analysis and clustering analysis of DEGs were performed using R version 4.3.2 packages. GO and KEGG functional enrichment analysis were performed to screen DEGs related to the biosynthesis and accumulation of sesquiterpenoids.

### 4.6. Quantitative Real-Time Polymerase Chain Reaction Analysis

The total RNA of A.M. was extracted using the UNIQ-10 column-type Trizol Total RNA Extraction Kit (Sangon Biotech, Shanghai, China), following the manufacturer-provided operation protocol strictly. Primers were designed according to the primer design principles by employing Primer Premier 5.0 software (detailed in Table 4). Based on the Ct values of the target gene, Ubiquitin, and the reference gene Ubiquitin, the ΔCt and ΔΔCt values of the experimental group and the control group were derived. Finally, the 2^−(ΔΔCt)^ value was calculated to validate the expression of the four DEGs involved in the MVA and MEP pathways [56].

### 4.7. High-Performance Liquid Chromatography Analysis of Terpenoid Compounds

Samples of A.M. from various origins were collected and analyzed by HPLC to determine the content of atractylenolide I, atractylenolide III, and atractylenolide. Standard compounds were purchased from Shanghai Yuanye Biotechnology Co., Ltd. (Shanghai, China). The extraction methods and procedures followed the guidelines specified in the *Chinese Pharmacopeia* (2020 edition) [5]. Analysis of A.M. extracts was performed using a Waters 2695 HPLC system equipped with a Waters 2998 photodiode array detector (Milford, MA, USA). The chromatographic column was a Waters Sunfire C18 (4.6 mm × 250 mm, 5 μm). Specific chromatographic conditions are as follows: column temperature 35 °C, mobile phase acetonitrile (A): water (B), gradient elution (0–10 min, 59% A, 10–25 min, 59% A~95% A, 25–35 min, 59% A, 35–40 min, 59% A), flow rate 0.8 mL/min, detection wavelengths 220 nm and 275 nm, and injection volume 10 μL.

## 5. Conclusions

In this study, we conducted a transcriptome sequencing and HPLC analysis of A.M. rhizomes from different origins. Eight key differentially expressed genes encoding enzymes in the MVA and MEP pathways were identified, among which *ISPF*, *GCPE*, *HMGCR*, and *FDPS* were significantly upregulated in Shaanxi samples; the accumulation of atractylodin, atractylenolide I, and atractylenolide III was also the highest. In contrast, A.M. from other regions showed lower expression of these biosynthetic genes and lower sesquiterpenoid contents. These results indicate that high expression of a single gene cannot drive efficient terpenoid biosynthesis, and breeding strategies should focus on coordinated regulation of multiple genes, especially the balanced optimization between upstream precursor supply and downstream skeleton formation.

In short, the differences in gene expression may be closely related to climatic conditions (temperature, light, and precipitation), soil characteristics (pH, mineral composition, and microbial community), genetic background, and cultivation practices. This finding provides a solid molecular biological basis for the study of the formation mechanism of authentic medicinal materials and the high-quality breeding of A.M. varieties.

## Figures and Tables

**Table 1:** Statistical table of transcriptome assembly results.

Categories	Transcripts	Unigenes
Total sequence number	915,152	374,475
Length ≥ 500 bp	390,703	128,529
Length ≥ 1000 bp	174,160	49,105
Min length	201	201
Max length	15,628	15,628
Total length	630,146,258	215,050,079
Average length	688.57	574.27
N50 length (bp)	1003	760
N90 length (bp)	760	256

**Table 2:** Differences between different local differential gene KEGG metabolic pathways.

Group	Top Five Class KEGG Pathway Names	Number
S vs. A	Ribosomes	77
	Carbon metabolism	52
	Starch and sucrose metabolism	43
S vs. F	Ribosomes	256
	Carbon metabolism	162
	Biosynthesis of amino acids	121
S vs. H	Ribosomes	200
	Oxidative phosphorylation	110
	Carbon metabolism	117
A vs. H	Ribosomes	60
	Protein processing in the endoplasmic reticulum	38
	Carbon metabolism/RNA transport	36
F vs. H	Oxidative phosphorylation	67
	Carbon metabolism/Neurotrophin signaling pathway	62
	Ribosomes	54
F vs. A	Ribosomes	144
	Starch and sucrose metabolism	78
	Carbon metabolism	76

**Table 3:** Comparison of key enzyme gene expression differences in terpenoid biosynthesis pathways of A.M. from different origins.

Key Enzymes	Gene Annotation Information	A vs. H	F vs. A	F vs. H	S vs. A	S vs. F	S vs. H
*DXS*	TRINITY_DN98338_c5_g1	15.53	−6.54	——	−15.53	——	——
	TRINITY_DN118127_c2_g1	——	15.75		15.17	——	——
***ISPF***	**TRINITY_DN102344_c2_g1**	3.67	——	3.87	——	——	3.39
	TRINITY_DN113213_c0_g5	——	——	——	——	−16.44	——
*ISPS*	TRINITY_DN113106_c1_g2	−6.0	——	——	6.23	——	——
	TRINITY_DN121289_c2_g5	4.23	−5.32	——	−4.7	——	——
	TRINITY_DN121289_c2_g1	4.94	−6.04	——	−5.42	——	——
	TRINITY_DN121289_c3_g2	4.77	——	——	−4.59	——	——
	TRINITY_DN103012_c0_g1	——	8.63	4.34	6.94	——	——
	TRINITY_DN124089_c3_g2	——	−5.44	——	——	——	——
*ISPE*	TRINITY_DN114236_c2_g1	——	3.44	——	——	——	——
***GCPE***	**TRINITY_DN119478_c1_g1**	——	——	4.55	——	——	5.33
***FDPS***	TRINITY_DN58900_c0_g1	−13.6	——	−7.22	——	——	——
	**TRINITY_DN108354_c1_g1**	——	4.6	5.19	4.55	——	5.15
	TRINITY_DN90804_c0_g1	——	——	——	−3.89	——	——
	TRINITY_DN88578_c0_g1	——	——	——	——	——	−12.23
*ACAT*	TRINITY_DN120516_c1_g3	——	——	——	6.69	3.82	——
	TRINITY_DN92952_c0_g1	——	——	——	——	−6.18	——
	TRINITY_DN90745_c0_g2	——	——	——	——	−12.82	——
***HMGCR***	TRINITY_DN100174_c0_g1	——	——	——	−3.36	——	——
	**TRINITY_DN112315_c0_g2**	——	——	——	14.5	14.5	14.5
*MVD*	TRINITY_DN102480_c1_g1	——	——	——	3.37	——	——
*PCME*	TRINITY_DN105029_c1_g2	——	——	——	−4.28	——	——
	TRINITY_DN90804_c0_g1	——	——	——	−3.98	——	——
*STE24*	TRINITY_DN87551_c0_g1	——	——	——	——	−4.11	——
*FNTB*	TRINITY_DN110526_c2_g2	——	——	——	——	——	14.1

Positive values indicate upregulation, negative values indicate downregulation, and values in bold represent significantly differentially expressed genes, and “——“ indicates no significant differential expression or undetected gene expression in the corresponding comparison group.

**Table 4:** Sequence of primers used in qRT-PCR.

Gene	Forward Primers	Reverse Primers
Ubiquitin	GATTTGCATACCACCACGAAG	AAGTAGAAAGCTCCGACACCAT
*IPSE*	GGACTACTTCTTTGATGAAAACTGA	TTTGGGTCTACCTGACACTGG
*GCPE*	GTGGGTGTTGTTTGAACTGCTC	ACAGACCCGAAATAAAGAACCTC
*HMGCR*	AACACCCTCACACCAGCAGAT	GGACCCTGCCCAAAACATC
*FDPS*	CTGCAACAATGAGCACCGAT	ACCAACCAAGGGCACACG

## Data Availability

All essential data supporting the key findings of this study have been included in the submitted manuscript. No additional datasets were generated or analyzed beyond those presented herein. Due to restrictions on the ownership and management of medicinal plant research resources, the authors do not hold the authorization to make the original research data publicly available, and there are no publicly archived datasets associated with this study.

## Abbreviations

The following abbreviations are used in this manuscript:

A.M.	*Atractylodes macrocephala*
bp	base pair
qRT-PCR	quantitative real-time polymerase chain reaction
HPLC	high-performance liquid chromatography
BLAST	Basic Local Alignment Search Tool
GO	gene ontology
NR	NCBI non-redundant protein database
CDD	Conserved Domain Database
PFAM	Protein Families Database
KOG	euKaryotic Orthologous Groups
Swissprot	Swiss-Prot Protein Sequence Database
NT	Nucleotide Sequence Database
KEGG	Kyoto Encyclopedia of Genes and Genomes
DEGs	differentially expressed genes
MVA	mevalonate
MEP	methylerythritol phosphate
IPP	isopentenyl pyrophosphate
FPP	farnesyl pyrophosphate

## Appendix A

**Table A1:** Statistical table of sequencing data quality preprocessing.

Sample	Raw Reads	Raw Bases (bp)	Total Clean Reads	Clean Bases (bp)	GC Base Ratio%	Q20 (%)	Q30 (%)
A1	58,513,266	8,776,989,900	56,554,110	8,083,340,053	46.90%	98.68	95.09
A2	48,741,824	7,311,273,600	47,615,740	6,914,157,200	46.41%	98.79	95.39
A3	68,116,068	10,217,410,200	66,441,512	9,645,298,208	46.44%	98.78	95.38
F1	66,118,908	9,917,836,200	63,846,718	8,967,518,770	46.07%	98.71	95.17
F2	61,448,460	9,217,269,000	59,914,550	8,632,686,915	46.24%	98.78	95.37
F3	66,995,486	10,049,322,900	64,339,162	9,144,040,803	46.44%	98.59	94.81
H1	62,200,254	9,330,038,100	60,835,706	8,836,916,202	45.20%	98.89	95.68
H2	67,874,028	10,181,104,200	66,255,006	9,575,996,019	45.12%	98.73	95.20
H3	66,325,174	9,948,776,100	64,629,984	9,366,665,180	44.98%	98.80	95.40
S1	61,571,838	9,235,775,700	60,117,158	8,719,798,400	46.40%	98.75	95.29
S2	76,311,598	11,446,739,700	74,287,334	10,739,613,623	46.40%	98.72	95.18
S3	74,683,862	11,202,579,300	72,738,186	10,514,272,054	46.49%	98.73	95.22

Q20 (%): the percentage of bases with an accuracy of more than 99% (the proportion of mass values ≥ 20); Q30 (%): The percentage of bases with a base recognition accuracy of more than 99.9% (the percentage of bases with a mass value of ≥30).
